# Getting fit for hip and knee replacement: a protocol for the Fit-Joints pilot randomized controlled trial of a multi-modal intervention in frail patients with osteoarthritis

**DOI:** 10.1186/s40814-018-0316-2

**Published:** 2018-07-20

**Authors:** Ahmed M. Negm, Courtney C. Kennedy, George Ioannidis, Olga Gajic-Veljanoski, Justin Lee, Lehana Thabane, Jonathan D. Adachi, Sharon Marr, Arthur Lau, Stephanie Atkinson, Danielle Petruccelli, Justin DeBeer, Mitchell Winemaker, Victoria Avram, Benjamin Deheshi, Dale Williams, David Armstrong, Barry Lumb, Akbar Panju, Julie Richardson, Alexandra Papaioannou

**Affiliations:** 1 Geriatric Education and Research for the Aging Sciences (GERAS), St Peter’s Hospital, 88 Maplewood Ave, Hamilton, ON L8M 1W9 Canada; 2 School of Rehabilitation Sciences, IAHS 403, McMaster University, 1400 Main Street West, Hamilton, ON L8S 1C7A Canada; 3 Department of Medicine, McMaster University, 1280 Main St West, Hamilton, ON L8S 4L8 Canada; 4 Department of Health Research Methods, Evidence, and Impact, McMaster University, 1280 Main St West, Hamilton, ON L8S 4L8 Canada; 5 Department of Pediatrics, McMaster University, 1280 Main St West, Hamilton, ON L8S 4L8 Canada; 6 Biostatistics Unit, St Joseph’s Healthcare—Hamilton, 50 Charlton Avenue East, Hamilton, Ontario L8N 4A6 Canada; 7 Department of Surgery, Division of Orthopaedics, McMaster University, 1280 Main St West, Hamilton, Ontario L8S 4L8 Canada

**Keywords:** Feasibility, Frailty, Phenotype, Fried, Short performance physical battery (SPPB), Hip replacement, Knee replacement, Rehabilitation, Exercise, Geriatrics

## Abstract

**Background:**

Joint replacement provides significant improvements in pain, physical function, and quality of life in patients with osteoarthritis. With a growing body of evidence indicating that frailty can be treated, it is important to determine whether targeting frailty reduction in hip and knee replacement patients improves post-operative outcomes.

**Objectives:**

The primary objective is to examine the feasibility of a parallel group RCT comparing a preoperative multi-modal frailty intervention to usual care in pre-frail/frail older adults undergoing elective unilateral hip or knee replacements. The secondary objectives are

1. To explore potential efficacy of the multi-modal frailty intervention in improving frailty and mobility between baseline and 6 weeks post-surgery using Fried frailty phenotype and short performance physical battery (SPPB) respectively.
2. To explore potential efficacy of the multi-modal frailty intervention on post-operative healthcare services use.

**Methods/Design:**

In a parallel group pilot RCT, participants will be recruited from the Regional Joint Assessment Program in Hamilton, Canada. Participants who are (1) ≥ 60 years old; (2) pre-frail (score of 1 or 2) or frail (score of 3–5; Fried frailty phenotype); (3) having elective unilateral hip or knee replacement; and (4) having surgery wait times between 3 and 10 months will be recruited and randomized to either the intervention or usual care group. The multi-modal frailty intervention components will include (1) tailored exercise program (center-based and/or home-based) with education and cognitive behavioral change strategies; (2) protein supplementation; (3) vitamin D supplementation; and (4) medication review. The main comparative analysis will take place at 6 weeks post-operative. The outcome assessors, data entry personnel, and data analysts are blinded to treatment allocation. Assessments: feasibility will be assessed by recruitment rate, retention rate, and data collection completion. Frailty and healthcare use and other clinical outcomes will be assessed. The study outcomes will be collected at the baseline, 1 week pre-operative, and 6 weeks and 6 months post-operative.

**Discussion:**

This is the first study to examine the feasibility of multi-modal frailty intervention in pre-frail/frail older adults undergoing hip or knee replacement. This study will inform the planning and designing of multi-modal frailty interventional studies in hip and knee replacement patients.

**Trial registration:**

ClinicalTrials.gov NCT02885337

**Electronic supplementary material:**

The online version of this article (10.1186/s40814-018-0316-2) contains supplementary material, which is available to authorized users.

## Background

Osteoarthritis (OA) is one of the most common chronic conditions worldwide and a major cause of morbidity, physical limitation, disability, and health care utilization [1, 2]. In 2010, the aggregate cost of managing OA in Canada was $10.2 billion [3]. It has been demonstrated that joint replacement provides significant improvements in pain, physical function, and quality of life in patients with OA [4, 5]. In Canada, during 2015–16, there were approximately 53,000 hip replacements and 64,000 knee replacements representing a 5-year increase of 18.1 and 15.7 5%, respectively [6]. It is expected that the number of older adults seeking total joint replacement will continue to rise [7].

Frailty is common in patients undergoing joint replacement [8] and refers to a medical syndrome characterized by “diminished strength, endurance, and reduced physiologic function” and with multiple causes and contributors [9]. Pre-frailty is an intermediate stage between non-frail and frail. Adverse outcomes associated with frailty include increased risk for functional disability, hospitalization, fractures [10], admission to long-term care, and increased mortality [11–14]. Frail older adults undergoing surgery are also more vulnerable to peri-operative stressors and are at increased risk of post-operative complications, increased length of stay, and discharge to assisted living [15, 16]. Recently, the Society for Perioperative Assessment and Quality Improvement recommended preoperative frailty screening evaluation and management [17]. With a growing body of evidence indicating that frailty can be treated [9] and improved [18], there is a need to examine whether targeting frailty reduction can improve the outcomes of pre-frail or frail adults who are undergoing joint replacement surgery.

Preparing patients for hip or knee replacement surgery through a prehabilitation model should be an integral part of the surgical care [19]. A recent systematic review and meta-analysis [20] examined the impact of preoperative physiotherapy on recovery after hip or knee joint replacement. Wang et al. pooled data from 22 RCTs (*N* = 1492 patients, mean age ranged from 51 to 76 years) and found that exercise/education slightly reduced pain scores within 4 weeks postoperatively and improved scores on the Western Ontario and McMaster Universities Arthritis Index at 6–8 and 12 weeks. There was no difference in SF-36 scores, length of stay, and total cost [20]. Another systematic review and meta-analysis [21] aimed to determine the effect of pre-operative interventions (exercise with or without education program) in patient waiting for hip and knee replacement. Wallis et al. included 23 RCTs (*N* = 1461 patients, mean age is 67.2 years) and concluded that exercise reduced pain for patients waiting for hip or knee replacement, and exercise with education programs improved activity after hip replacement [16].

Potential limitations of all the previous RCTs that examined pre-hip or knee replacement interventions include (1) none of these studies identified prefrail or frail population, (2) none used multi-modal interventions, (3) the duration of the interventions ranged between 2 and 8 weeks in length (a longer intervention period may improve post-operative and long-term outcomes), (4) most participants were waiting for only knee (not hip) replacement, (5) most studies were at high risk of bias. For example Wang et al. included 18 out of 22 studies with high risk of bias [20], thus, high quality RCTs are needed, and (6) most of the studies did not report critical outcomes such as frailty and treatment adherence [20].

Since, frail individuals are at greater risk of post-operative complications [15, 16], it is important to implement strategies to improve the “fitness” of frail patients pre-operatively. While previous studies with single interventions have demonstrated some effectiveness [15], multi-modal approaches have not been examined in individuals undergoing joint replacement surgery. International consensus guidelines [9] recommended an evidence-based multi-modal approach (including exercise, protein-calorie supplementation, vitamin D, and reduction of poly-pharmacy) to target frailty pre-operatively. The proposed study is a pilot RCT comparing a pre-operative multi-modal frailty intervention to usual care among pre-frail/frail patients undergoing unilateral elective total hip or knee replacement surgery. The current report outlines the research design and protocol for evaluating this pilot RCT.

### Theory and development framework

**Fig. 1:**
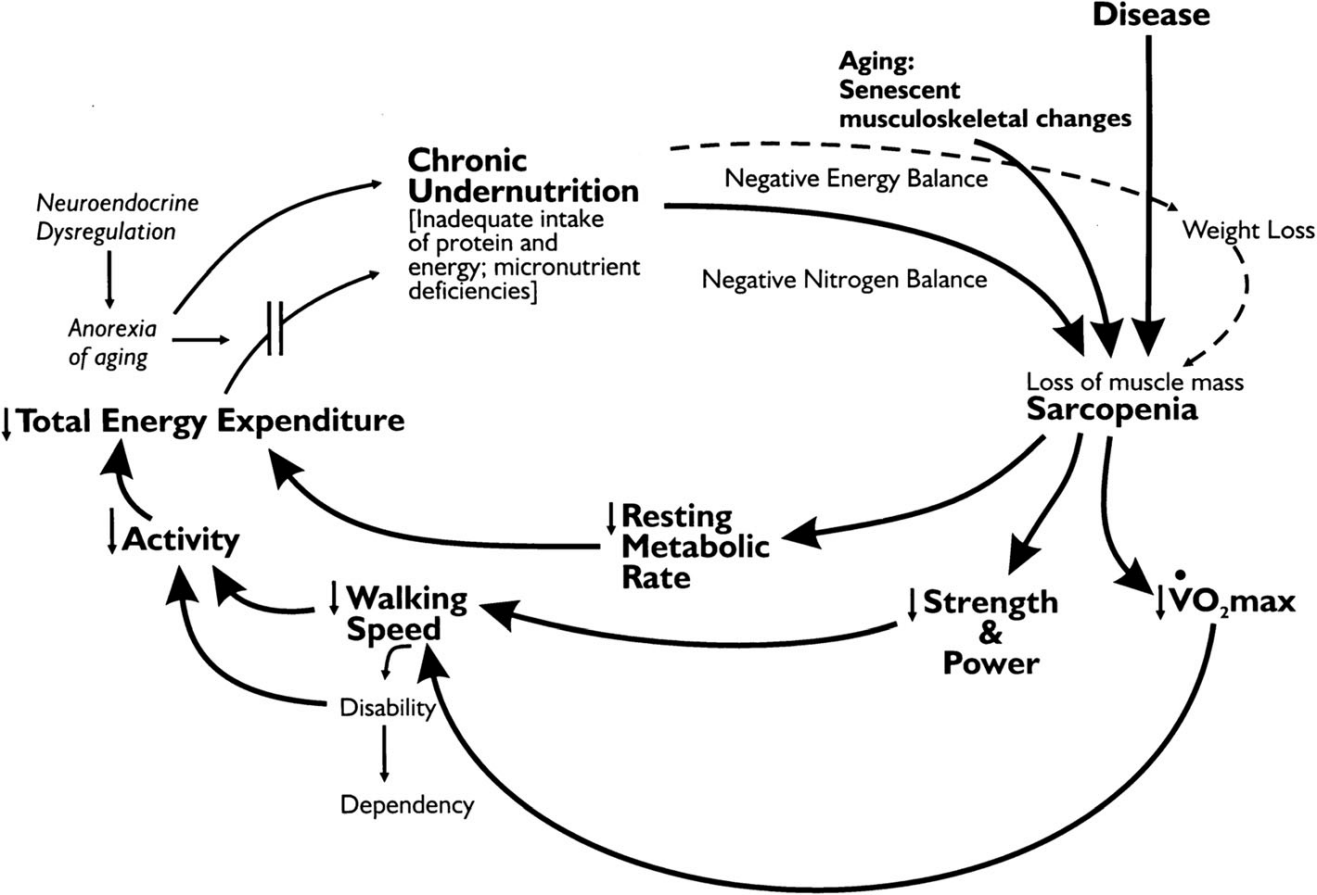
Cycle of frailty

The cycle of frailty model proposed by Fried et al. 2001 (Fig. 1), identified key elements of frailty [11]. The core elements of the Fried frailty cycle incorporated the main frailty markers, including age-associated declines in lean body mass, strength, endurance, balance, walking performance, and low activity. The proposed intervention components aim to improve all the frailty markers of the Fried frailty cycle [11]. As this is the first study to implement preoperative multi-modal intervention, a pilot study is required to assess the fidelity of intervention delivery and the feasibility of (1) study process (recruitment and retention rate), (2) study resources (required time and budget), (3) management (study personal and data management), and (4) scientific (treatment safety, and estimation of potential treatment effect and its variance) [22].

### Objectives

The primary objective is to examine the feasibility of a parallel group RCT comparing a preoperative multi-modal frailty intervention to usual care in pre-frail/frail older adults undergoing elective unilateral hip or knee replacement. The secondary objectives are

1. To explore potential efficacy of the multi-modal frailty intervention in improving frailty and mobility between baseline and 6 weeks post-surgery using Fried frailty phenotype and short performance physical battery (SPPB), respectively.
2. To explore potential efficacy of the multi-modal frailty intervention on post-operative healthcare services use including hospital length of stay, rate of complication after hip or knee replacement, readmission to the hospital, and number of emergency room (ER) visits.

## Methods/Design

### Study design

Fit Joints study is a pilot parallel group RCT comparing a 3–10 months, pre-operative multi-modal frailty intervention, and usual care among pre-frail/frail patients undergoing total hip or knee replacement surgery.

The main group comparisons will occur at 6 weeks post-operative. Both groups will also be assessed at 6 months post-operative. The trial has been registered with Clinical

**Fig. 2:**
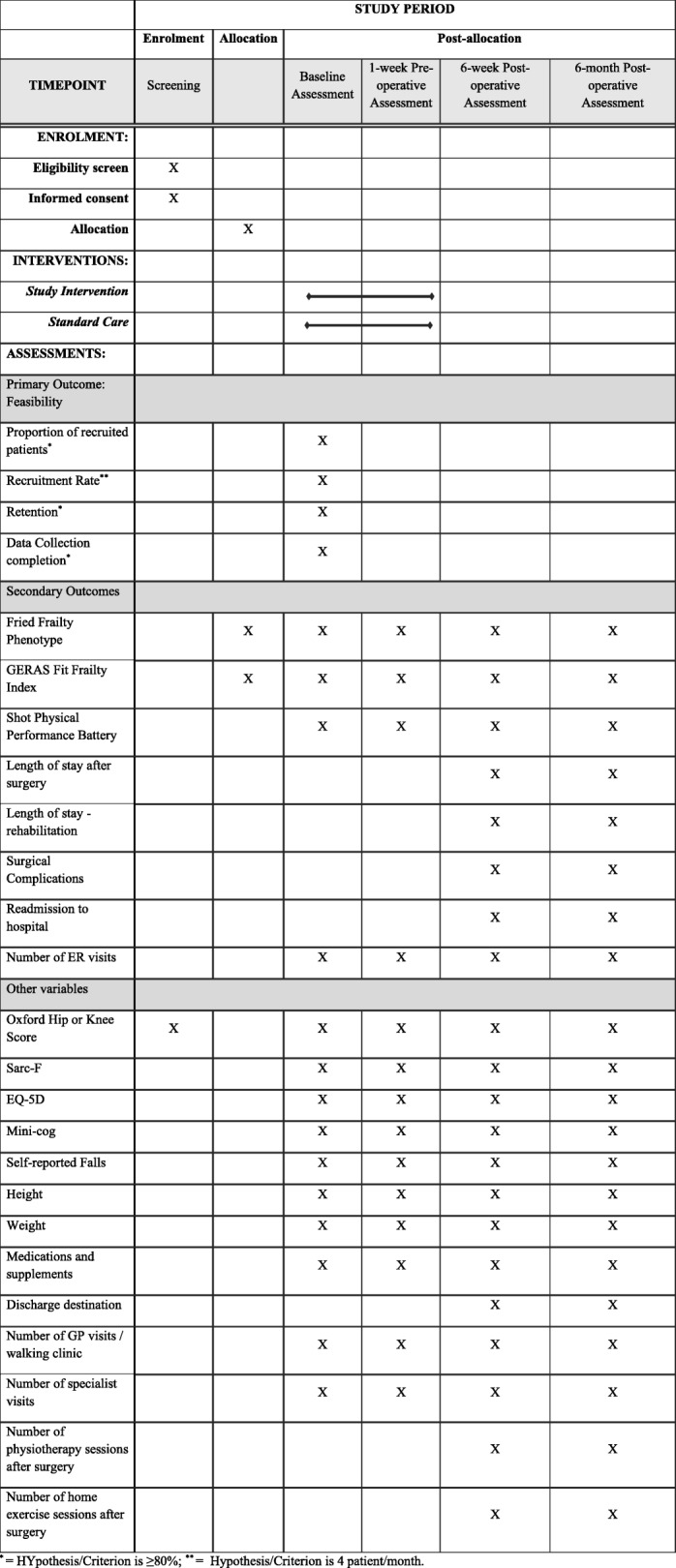
Standard Protocol Items Recommendations for Interventional Trials (SPIRIT) Schedule of enrolment, interventions, and assessments

Trials.gov NCT02885337. We used the Standard Protocol Items: Recommendations for Interventional Trials (SPIRIT) guidelines to guide the reporting of our trial protocol [23]. A SPIRIT Checklist is provided as Additional file 1, and a flow diagram is included as Fig. 2.

### Study setting

We are recruiting participants from the Regional Joint Assessment Program [24] (RJAP) at a tertiary care academic hospital (Juravinski Hospital) of Hamilton Health Science—Hamilton, Ontario, Canada. RJAP program serves patients with arthritis referred from primary care physicians to be assessed for hip or knee joint replacement by advanced practice physiotherapists (APPs) (physiotherapist with a special orthopedic training) and orthopedic surgeons [24]. After recruitment, the intervention will take place in a community setting, including the participant’s home and community centers.

### Eligibility criteria

Participants will be included if they are (1) ≥ 60 years old; (2) pre-frail (score of 1 or 2) or frail (score of 3–5; Fried frailty phenotype [11]); (3) receiving elective unilateral hip or knee replacement; and (4) waiting time to surgery is estimated to be between 3 and 10 months. Participants will be excluded if reported as having (1) renal insufficiency (due to potential contraindication of additional protein); (2) neuromuscular disorder; (3) active cancer; or (4) any inflammatory arthritis.

### Recruitment strategy

After the APPs and orthopedic surgeons assess patients referred for hip or knee problems, APPs will explain the study, invite potential participants, and screen them for eligibility. A research assistant will help the APPs in administering the Fried frailty phenotype. If eligible participants are considering the study and need time to decide, they receive the study information sheet and will be contacted by a research assistant to confirm their participation. The clinic administrations (who are blinded to the patient participation in the study) place the patients in the surgery wait list and assign them a surgery date later.

### Randomization and consent

Once the study research assistant confirms the patient’s eligibility, and obtains informed written consent, the research assistant will submit the eligibility form, consent form, and participant contact form to a team member (who is not part of the study) who will randomize the participant to the intervention or usual care group based on stratified block randomization list. To ensure an equal number of participants in the study groups, the allocation ratio will be 1:1. Participants will be stratified based on their age (≥ 80 or ≤ 79 years) and approximate waiting time (≥ 6 or < 6 months). The allocation sequence will be computer generated using SAS 9.3 software [25]. To conceal the sequence, only a researcher who is not involved in the study will have the computer-generated allocation list.

After randomization, a blinded outcome assessor will contact the participants to set up an appointment for the baseline assessment. After the baseline assessment, the study research coordinator will inform the participants of their study group. The intervention group participants will be contacted by the study intervention kinesiologist to arrange the first intervention visit.

Those blinded to the intervention will include the outcome assessors who conduct assessments at the RJAP and in participant homes, the clinic administration, data entry personnel, data analysts who performs the final data analysis, the investigative team, and members of the steering committee. The patient will also be blinded at the baseline assessment. The study coordinator, study intervention kinesiologist, and participants will not be blinded.

### Development and piloting the Fit Joints intervention

Due to the complexity of the frailty syndrome, we are developing the proposed multi-modal frailty intervention using the revised Medical Research Council framework for design and evaluation of complex interventions [26, 27]. The FIT trial in Australia demonstrated successful frailty reduction after implementing a 1-year frailty intervention tailored to each participant based on comprehensive geriatric assessment. The target cohort was frail patients (three or more Fried criteria) seen in an aged care service [18].

### Multi-modal Fit Joints intervention components

**Fig. 3:**
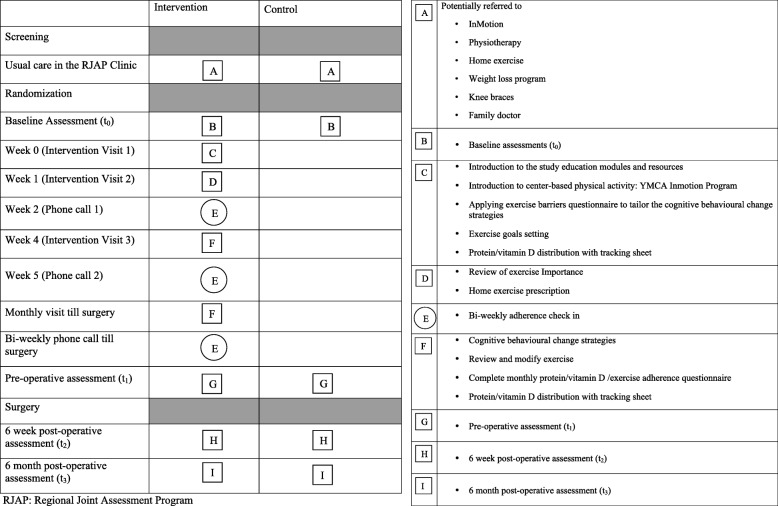
Study intervention and outcome assessments. The square means a visit and the circle means a phone call

The intervention and outcome assessment visits are summarized in Fig. 3.

Participants in the intervention group will receive, for up to 10 months, a multimodal program intended to target frailty reduction (as described in Table 1) between randomization and their surgery. The study intervention kinesiologist will manage the coordination of the exercise components of the intervention, and deliver vitamin D and protein supplementation. The study geriatrician will provide the medication review component, and 2 of the study investigators who are expert in nutrition will review the vitamin D and protein supplementation.

**Table 1:** Components of the multimodal intervention

Component	Dose/material provided	Description
Exercise and coaching	Based on Canada’s Physical Activity Guidelines^1^ Minimum: 3× week for 45–60 min at home and/or YMCA [28, 44]	• Kinesiologist assessment: goal setting, cognitive behavior change strategies^2^ • HOME: tailored exercise program based on individual ability and exercise preference (i.e., chair versus standing exercises). Functional movements to mimic ADL’s (i.e., getting up from a chair). Exercise bands will be provided. • All participants will progress based on their current physical activity levels while focusing on personal fitness and health goals set at the beginning of the program. • YMCA: InMotion program • GOAL: endurance, resistance, and balance training 3× week for 45–60 min at home and/or YMCA • Monthly kinesiologist visit/bi-weekly phone calls [28]. • Participants will track their exercise in a study-tracking logbook.
Protein	1–2 Ensure Enlive™ protein daily	• Each serving (vanilla or chocolate flavor) contains 350 kcal, 20 g protein, 1.5 g β-Hydroxy β-Methylbutyrate (HMB) • Advised to take the protein supplement with a meal or within 3 h of exercise on activity days [32, 33]. • Pre-albumin serum level tested at the screening and 6-week postoperative visits (carried out by the clinic nurse during these visits).
Vitamin D	1 × 1000 IU/day, unless prescribed otherwise by family physician.	• Vitamin D3 (1000 IU tablets) • Serum 25 (OH). Vitamin D serum level tested at the screening and 6-week postoperative visits.
Medication review		• A pharmacist trained geriatrician (Dr. Lee) will review the medications of participants in the intervention arm using subsets of Beers [45] and STOPP/START criteria [46] to check for any inappropriate medications. • Any recommendations will be mailed/faxed to the participants’ family physicians for their consideration by the central site coordinator.

^1^Based on Canadian Physical Activity Guidelines for those aged 65 years or older which recommend cardiorespiratory, strength, balance, and flexibility exercise components [25]

^2^Topics to support patients in achieving their health goals could include (1) goal setting, (2) self-monitoring, (3) time management, (4) overcoming barriers, (5) environmental scan, (6) social support, and (7) stimulus control

### Kinesiologist visit schedule and delivery of coaching/supplements

After randomization, the study intervention kinesiologist will phone the participant to book a home visit where goal setting will be done. During months 2–10, the study intervention kinesiologist will have bi-weekly contact (one monthly visit and one phone-call in the interim) [28] to check on progress. At the visits, the kinesiologist will adjust programs as needed, provide ongoing coaching/education, and deliver vitamin D/protein supplements. We will use exercise-reporting guidelines (CERT) to guide reporting the exercise component of the intervention [29]. Participants will be encouraged to use the Borg Rating of Perceived Exertion 10-point Scale to monitor their perceived effort levels and exhaustion for each exercise component (1 means rest/no effort and10 means maximal effort) [30]. Participants will be asked to work in a 5–7/10 workload (i.e., 50–60% of their maximal heart rate). The Borg Scale will help participants to work based on how they are feeling, which is safer for the geriatric population. In the first intervention visit, the study kinesiologist will ask the participants if they would prefer to do center-based or home-based exercise, or both.

### Center-based exercise

If a participant decides to do center-based exercise, they will be provided with a free membership of the YMCA community center to participate in the “InMotion program”. This community-based program is designed for people with chronic bone and joint health problems such as osteoporosis and arthritis. It is also appropriate for those wanting to improve their health before and after having hip or knee replacement surgery. Fitness trainers lead the InMotion program, and if needed, an experienced physiotherapist is available for consultation. The program includes hydrotherapy, aerobic exercise, and 12 education sessions. Participants will be encouraged to attend the InMotion program components at least three times per week. Additional file 2 shows further description to the Fit Joints exercise.

### Cognitive behavioral change strategies (CBCS)

The participant’s readiness to exercise and their exercise barriers/facilitators will be determined using a self-reported questionnaire guided by the trans-theoretical model of behavior change (TTMBC) [31, 32]. Based on the participant’s readiness to exercise, CBCS may be an effective way to promote exercise in older adults [33]. In the current study, the administration of the modules will be dependent on the challenges that the participants express using the exercise barrier/facilitators questionnaire (i.e., lack of time, motivation, social support). These strategies are based on the TTMBC [31, 32]. The 7 CBCS topics that will be incorporated over the intervention period will include (1) goal setting: to assist with the development of their tailored exercise program, (2) self-monitoring: to track their exercise progress/goals/behavior, (3) time management strategies: to find more time to exercise, (4) overcoming barriers: to overcome adversity in exercise routines, (5) environmental scan: to help participants identify local/available resources and support, (6) social support: to find participants’ support system to achieve physical activity, and (7) stimulus control: to create participants’ planned reminders for increasing physical activity.

### Control group

Patients in the control group will receive usual care, which may include recommendations from their orthopedic surgeon to attend exercise programs, fitness and educational classes, physiotherapy referral, pool therapy, or weight loss program before surgery. However, these patients will not receive any support from the study intervention kinesiologist. Participants in the control and intervention groups will be instructed to complete a dietary intake log (including days of the week and weekend days) that indicates the type of food and amount ingested over a 4-day period in order to calculate energy and micronutrient consumption.

### Study outcomes

#### Primary outcome: feasibility

Feasibility will be assessed by (1) recruitment rate, (2) retention rate, and (3) data collection completion [22]. Figure 2 summaries the primary and secondary outcomes and measurement time. The Fit Joints intervention fidelity (the degree to which the Fit Joints intervention is delivered) will be assessed by measuring the length of the intervention and number of intervention components delivered by the study kinesiologist. We will measure participants’ adherence to each component of the intervention (center-based or home exercise, protein and vitamin D supplement, and medication review). Adherence will be measured by a monthly self-reported form developed specifically for the Fit Joints trial.

#### Secondary outcomes

Frailty will be assessed using (1) Fried frailty phenotype which is composed of five items, three self-reported (unintentional weight loss, exhaustion and physical activity), and two performance-based items (strength (assessment based on the handgrip strength measurement) and gait speed). It is a widely used and validated frailty measure [11, 34]. Each item is scored 0 or 1 with a final score out of 5; higher scores indicate greater frailty; and (2) short performance physical battery (SPPB) (made up of three assessments [35]: (a) the 4-m walk test (walking speed); (b) chair rise: balance and coordination (the ability to rise from a chair without arms); and (c) the standing balance test). The participant is evaluated on each assessment using a score between 0 and 4. A final summary performance score out of 12 is calculated, with higher scores indicating superior lower extremity function [35]. The SPPB has also been validated and has demonstrated good internal consistency and responsiveness [36, 37]. Healthcare service use (including patients’ medications/supplements (dose, frequency, and duration), discharge destination, length of hospital stay, rehospitalization rate, number of visits to general practitioner, emergency room, specialists, and physiotherapist, and number of home exercise sessions) will be collected using a form specifically developed for this study. Other outcomes listed in Fig. 2 will be collected. The study outcomes will be collected at the baseline, 1 week pre-operative, and 6 weeks and 6 months post-operative.

### Adverse events

Adverse events or harm from any source will be reported to the research team and recorded on a structured form. Any serious adverse events will be reported to the Research Ethics Board within 24 h. Participants will be instructed to contact the study coordinator if they experience any unfavorable/unintended signs or symptoms. An independent Data Safety and Monitoring Committee will review safety data from the trial and advise the investigators and the Steering Committee on the future management of the trial.

### Data collection and management

Figure 2 provides an overview of the data collection timeline. The baseline and 1-week preoperative assessments will be conducted in the participant’s home, and the 6-week and 6-month assessments will be conducted in the orthopedic clinic. All four assessments will be conducted by blinded assessors. The study assessors received an individualized 3-day training on how to collect the study outcome measures from frail older adults. Study data will be managed using REDCap electronic data capture tools [38]. The study database will be password protected and kept on a secure network system.

### Trial management

The coordinating center for the study is at the Geriatric Education and Research in Aging Sciences (GERAS) Center, Hamilton Health Sciences. The study coordinator and research assistants will be responsible for submitting and maintaining REB documents, scheduling of home visits, receiving, and storing consent forms. The study Steering Committee will meet every 6 months to provide overall supervision of the trial. The research coordinator will call more frequent Steering Committee meetings if required. It is anticipated the final results of this study will be completed in 2018.

### Data analysis

Data from the trial will be analyzed and reported in accordance with the CONSORT criteria [33, 39]. The baseline characteristics will be reported as mean (standard deviation) or median (inter-quartile range) values for continuous variables and as counts (percent) for categorical variables. Data will be summarized in tabular or graphical form. The main between-group comparison will take place at 6 weeks post-operative. The primary feasibility outcomes will be analyzed using descriptive statistics expressed as percent and corresponding 95% confidence intervals (CI). For clinical outcome, analyses will be performed using the intention-to-treat principle. We will use linear regression for continuous variables and logistical regression for categorical variables to explore the difference between groups pre- and post-operative. Exploratory subgroup analyses will be conducted to explore the differential effect of home-based versus center-based exercise and the effect on people undergoing hip versus knee replacement. Sensitivity analysis will be conducted using the per-protocol concept (including adherent participants, who completed 70% of the intervention components (i.e., completed 70% of exercise sessions, took 70% of the vitamin D, protein supplements, and the medication review was done)) [40]. All *p* values will be reported to three decimal places with those less than 0.001 reported as *p* < 0.001. The criterion for statistical significance will be set a priori at alpha = 0.05. Analyses will be performed using STATA V13 [41].

### Sample size

The sample size calculation was conducted using PASS software (Kaysville, UT) and was based on the feasibility outcomes of 80% for screening, retention, and data completion [33]. We will need a sample size of 62 participants to produce a two-sided 95% confidence interval with a width equal to ± 10% and an 80% criterion for success.

### Ethical considerations

The study was approved by the Hamilton Integrated Research Ethics Board (file # 2017-1565). Participants will undergo an informed consent process and sign a consent form prior to randomization.

## Discussion

This is the first study to examine the effect of multi-modal frailty intervention in frail and/or pre-frail older adults undergoing hip or knee replacement. We conducted a literature search in MEDLINE database using frailty, hip or knee replacement, and randomized controlled trial as keywords, and we did not find any multi-modal frailty intervention trials. Given this is a pilot study, we will learn about the feasibility of applying this multi-modal frailty intervention in people waiting for hip or knee replacement surgery.

The study intervention will increase the engagement of community resources (such as YMCA center-based exercise) by older adults, which will contribute to the older adults’ community participation and sustainability of the Fit Joints intervention. We hypothesize that a multi-modal intervention targeting exercise, vitamin D and protein supplementation, and a reduction of poly-pharmacy will synergistically improve pre- and post-operative frailty status and physical function in pre-frail/frail patients undergoing hip or knee replacement surgery. The results of this pilot trial will inform the design and implementation of a subsequent multi-center trial.

The duration of the Fit Joints intervention will vary according to the surgery waiting time, which addresses practical questions about the risks, benefits, and costs of an intervention as they would occur in routine clinical practice [42], rather than in an ideal setting. The Fit Joints study design emphasizes the contextual factors and real-world applicability of the study [43]. Also, Fit Joints intervention and outcomes are relevant to clinicians, patients, and decision-makers. Frailty is associated with higher complication rate; readmissions and longer hospital stay after hip or knee replacement surgery [8]. Carrying out the Fit Joints pilot trial is critical to see if a definitive multi-center trial can determine the effect of the Fit Joint intervention on pre-operative frailty, post-operative outcomes and complication, and health services use after hip or knee replacement surgery.

Medical Research Council criteria define a “complex intervention” as interventions that are built up from a number of components, which may act both independently and inter-dependently [44]. These components include behaviors, behavior parameters, and methods of organizing those behaviors, and they may have an effect at the individual patient level, organizational, or service level or population level (or all of these in some cases). As any complex intervention, the Fit Joints intervention has several articulating components (including center and/or home-based tailored exercise program, cognitive behavioral coaching, protein and vitamin D supplements, and medication review). It is a challenge to (1) standardize all these intervention components and (2) determine the contribution of each intervention component and any interaction between these components [25]. Phase 0 (choosing an intervention theoretical model) and phase 1(identify the intervention components and the supporting evidence) of the Medical Research Council framework have been completed. The proposed study represents phase 2 of the Medical Research Council framework (which is examining the feasibility of the intervention). After completing this pilot study, we will complete phase 3 (definitive study) and phase 4 (dissemination and implementation).

The proposed study has some limitations. Participant recruitment will take place within one hospital site, which may limit its generalizability to other hospital care settings. Fit Joints investigators have considered the challenge of applicability to other settings during the study protocol development. Participants will be offered to do center-based or home exercise; however, some participants may not have access to the center-based exercise due to various reasons such as lack of time or transportation. Also, the pre-operative assessments will occur in different time points for different participants due to the variable intervention duration and that might lead to heterogeneity of the intervention effect across participants.

Strengths of our proposed study include (1) valid and reliable patient reported measures were used; and (2) Fit Joints study engaged all key stakeholders in the process of implementation, including patients, interdisciplinary healthcare teams, community organizations, and researchers. Having each perspective will enhance the participants experience throughout the study course.

The lessons learned from this pilot RCT will be helpful when planning and designing future frailty studies and will provide a better understanding of pre-operative frailty and surgical outcome. This includes insights on the study implementation process (e.g., participants’ recruitment and retention), resources (time and budget issues), management (personnel and data management issues), and scientific evidence (effect sizes) [33].

### Trial status

Participants are currently being recruited. Recruitment will be completed approximately on April 2018.

## Additional files

Additional file 1: SPIRIT Checklist. (DOC 121 kb)

Additional file 2: Detailed description to the Fit Joints exercise. (DOCX 31 kb)

## Abbreviations

APPs: Advanced practice physiotherapists
CBCS: Cognitive Behavioural Change Strategies
CI: Confidence intervals
ER: Emergency room
GERAS: Geriatric Education and Research in Aging Sciences
RJAP: Regional Joint Assessment Program
SPIRIT: Standard Protocol Items: Recommendations for Interventional Trials
SPPB: Short performance physical battery
TTMBC: Trans-theoretical model of behavior change
