# Supplementary material for: Getting fit for hip and knee replacement: a protocol for the Fit-Joints pilot randomized controlled trial of a multi-modal intervention in frail patients with osteoarthritis
Source: Pilot Feasibility Stud. 2018 Jul 20;4:127. doi: 10.1186/s40814-018-0316-2 (PMC6053795; doi:10.1186/s40814-018-0316-2)
Supplement: Supplementary file 2 — Detailed description to the Fit Joints exercise. (DOCX 31 kb) [file 40814_2018_316_MOESM2_ESM.docx]

**Additional file 2**

**Fit-Joints Exercise Recommendations & Progressions**

Participants will be encouraged to progress on all components of exercise: cardiovascular, muscular strength, balance and flexibility. All exercises are prescribed based on the recommendations from the Canadian Physical Activity Guidelines for older adults 65+ (Tremblay, et al., 2011). Participants will be encouraged to use a rating of perceived exertion to monitor their perceived effort levels and exhaustion for each exercise component (Borg, 1982). All participants will progress based on their current physical activity levels while focusing on personal fitness and health goals set at the beginning of the program.

**Cardiovascular** – participants are encouraged to obtain 150 minutes of moderate to vigorous physical activity per week in bouts of ten minutes or more. Participants will progress in 3 ways: i. increase the number of minutes per week or session, ii. Increase the intensity (30%-70%), or iii. Increase the number of days performing the exercise per week.

**Muscular strength** – participants should complete a minimum of 2 days of strength training per week with at least one exercise per major muscle group (i.e. chest, back, etc.). To progress, participants will increase the number of repetitions and/or sets as well as increase the level of the resistance band (i.e., light, medium, heavy). Participants can also progress by increasing the number of days per week they complete the exercises.

**Balance** – participants will be encouraged to complete regular balance training, these types of exercises can be performed daily to prevent falls or loss of balance. Participants will progress by increasing the level of difficulty for each exercise. Participants may progress from a stationary exercise (i.e., standing in one spot) to a dynamic exercise (i.e., tandem walking). Further, participants can be challenged by closing their eyes, if they feel safe doing so.

**Flexibility** – participants will be encouraged to complete daily flexibility exercises to preserve range of motion. Participants will progress by increasing the number of days they partake in this component as well as how long they hold each stretch for. Lastly, participants can progress by increasing the number of stretches or major muscle groups involved.

References

Borg, G. A. (1982). Psychophysical bases of perceived exertion. *Med sci sports exerc*, *14*(5), 377-381.

Tremblay, M. S., Warburton, D. E., Janssen, I., Paterson, D. H., Latimer, A. E., Rhodes, R. E., ... & Murumets, K. (2011). New Canadian physical activity guidelines. *Applied Physiology, Nutrition, and Metabolism*, *36*(1), 36-46

|  | **F re** | **I re** | **T re** | **T re** |
| --- | --- | --- | --- | --- |
| Exercise Type* | **F**requency: Times/week | **I**ntensity: Rating of perceived exertion | **T**ype: Equipment | **T**ime: Duration/Sets |
| ***Cardio*** | **Beginner**  2-3x/week  **Moderate**  4-5x/week  **Challenging**  6+x/week | **Beginner**  3-4 RPE  **Moderate**  5-6 RPE  **Challenging**  7-8 RPE | **Beginner**  Walking, leisure Swimming, Cycling  **Moderate**  Brisk walking, pole walking, Aqua-Fit  **Challenging**  Jogging, Racquet sports, golf | **Beginner**  5 -10 mins  **Moderate**  10-30 mins  **Challenging**  30+ mins |
| ***Strength*** | **Beginner**  2x/week  **Moderate**  3x/week  **Challenging**  4-7x/week | **Beginner**  Yellow  **Moderate**  Green  **Challenging**  Red, Purple | **Beginner**  Seated exercises  **Moderate**  Standing exercise/functional exercises  **Challenging**  Dynamic standing exercises | **Beginner**  1 set of 15 reps  **Moderate**  1-2 sets of 8-15  **Challenging**  1-2 sets of 10-15 |
| ***Balance*** | **Beginner**  2-3x/week  **Moderate**  3-4x/week  **Challenging**  5-7x/week | **Beginner**  Level 1 – stationary  **Moderate**  Level 2 – eyes closed stationary  **Challenging**  Level 3 - dynamic | **Beginner**  Holding on to a chair/counter  **Moderate**  Without holding on to supports  **Challenging**  Without holding supports and | **Beginner**  5-10 secs  **Moderate**  10-20 secs  **Challenging**  30+ secs |
| ***Flexibility*** | **Beginner**  2-3x/week  **Moderate**  3-4x/week  **Challenging**  5-7x/week | **Beginner**  5 secs/stretch  **Moderate**  6-15 secs/stretch  **Challenging**  16-20 secs/stretch | **Beginner**  1 stretch per major muscle groups  **Moderate**  1-2 stretches  **Challenging**  3 stretches | **Beginner**  5-10 mins  **Moderate**  10-30 mins  **Challenging**  30+ mins |

Exercise Type* see Appendix 2 for descriptions.

**Participant Exercise Options**

|  | ***YMCA*** | ***Home*** | |
| --- | --- | --- | --- |
| ***Supervision & Qualifications*** | Certified Fitness Instructors, Registered Physiotherapist (Who instructs the Education Series). | Registered Kinesiologist with 5+ years’ experience with special populations and exercise prescription. | |
| ***Setting*** | Community, group exercise, class setting (~20-30 people/class) | Home setting (1-2 people if partner or family support) | |
| ***Consultation Points*** | Available to address questions/concerns at each exercise session | The Kinesiologist checks in once per month to review, progress, and/or change exercises. | |
| ***Specific/Tailored*** | Group environment, exercises are presented with exercise options (beginner or advanced) – participants are encouraged to complete the exercise intensity or challenge that is most appropriate for them. | Exercising on their own at home. Participants are encouraged to complete an exercise intensity that is appropriate for them, using pain as their guide (monitoring how the participant feels during and after exercise). | |
| ***Warm-up & Cool-down activities*** | 5-10 minutes to elevate heart rate. The goal is to increase overall ROM. Cool-downs will consist of a goal to bring heart rate back to normal (pre-exercise HR), in addition to static stretches of the major muscle groups. | | |
| ***Participant Exercise Familiarization/ Experience*** | If participants are new to the exercises they are supervised closely to ensure proper technique and safety. If exercises are too challenging or painful to complete, a modified movement to engage similar muscle groups will be demonstrated to the participant. | | |
| ***Exercise Order*** | Warm-up, main exercises, cool-down | | |
| ***Rest periods*** | 30 seconds to 1 minute will be encouraged between exercises. Depending on participant’s current fitness levels, rest times will be longer for beginners until cardiovascular capacity has increased. As participants progress, rest periods will be shorter in duration. | | |
| ***Baseline fitness levels*** | All participants are encouraged to slowly and cautiously progress with the amount of exercises they complete and to take breaks as needed. | | |
| ***Motivation & support*** | Encouraged and supported throughout the exercise sessions by fitness instructors. Instructors are always available for questions as needed by the participants. | | Participants are positively encouraged throughout the intervention with a monthly visit to review goals, and bi-weekly check-ins to address questions or concerns. |
| ***Adverse Event documentation and reporting*** | If an event occurs that is serious in nature, the YMCA records and follows YMCA safety and first aid protocols. | | Participants report all AE’s and falls to research assistants at monthly check points. |
